# Habitat imaging based on water–fat separation for interpreting extraocular muscle heterogeneity in thyroid eye disease

**DOI:** 10.1530/ETJ-25-0366

**Published:** 2026-04-17

**Authors:** Linhan Zhai, Yangyang Yin, Yu Chen, Weiqiang Liang, Feng Li, Qiuxia Wang, Gang Yuan, Jing Zhang

**Affiliations:** ^1^ Department of Radiology, Tongji Hospital, Tongji Medical College, Huazhong University of Science and Technology, Wuhan, Hubei, China; ^2^ Department of Radiology, Zhongnan Hospital of Wuhan University, Wuhan, Hubei, China; ^3^ Department of Radiology, Xiangyang Central Hospital, Affiliated Hospital of Hubei University of Arts and Science, Xiangyang, Hubei, China; ^4^ Department of Endocrinology, Tongji Hospital, Tongji Medical College, Huazhong University of Science and Technology, Wuhan, Hubei, China

**Keywords:** thyroid eye disease, extraocular muscle, habitat analysis, signal intensity, intravenous glucocorticoid

## Abstract

**Objective:**

The heterogeneity of extraocular muscles (EOMs) in thyroid eye disease (TED) correlates with response to intravenous glucocorticoid (IVGC) therapy but remains unquantified by current imaging. This study applied habitat imaging to characterize EOM heterogeneity and remodeling patterns.

**Methods:**

This retrospective study included 138 patients with active moderate-to-severe TED (84 responsive; 54 unresponsive), all undergoing water–fat separation imaging before and after IVGC therapy. Four EOM habitat subregions were defined using the Otsu method based on water and fat signal intensity (SI). Volume and volume percentage (VP), including their absolute (Δ) and relative (Δ%) changes post-therapy, were compared between groups. Univariate analysis compared habitat features, while binary logistic regression and receiver operating characteristic (ROC) curve analysis identified valuable parameters for evaluating therapeutic effect.

**Results:**

Habitat imaging identified edema (high SI_water_ and low SI_fat_) and myosteatosis (low SI_water_ and high SI_fat_) subregions. The responsive group exhibited a higher edema VP (32.64 vs 14.13%, *P* = 0.008) at baseline. After IVGC treatment, the responsive group had a significant decrease in edema VP (32.64 vs 9.85%, *P* < 0.001); both groups displayed increased myosteatosis, with more pronounced changes observed in the responders. The combination of Δ volume of myosteatosis region and Δ% volume of whole region demonstrated excellent discriminative ability (AUC = 0.86) with high sensitivity (93.75%) in treatment response assessment.

**Conclusions:**

Habitat imaging based on water–fat separation provides quantitative visualization of EOM pathological remodeling, establishing myosteatosis as an adjunctive biomarker for evaluating IVGC therapeutic response.

## Introduction

Thyroid eye disease (TED) is the most common orbital disease in adults and the leading extra-thyroid manifestation of Graves’ disease, characterized by orbital soft-tissue inflammation and affecting 25–50% of Graves’ disease patients (1, 2). Due to the strong anti--inflammatory properties, high-dose intravenous glucocorticoid (IVGC) therapy is recommended as the first-line treatment for active, moderate-to-severe TED (3). However, some TED patients show a poor response to IVGC, closely related to the heterogeneity of retrobulbar tissues (4). In TED, extraocular muscles (EOMs) and orbital fat are the primary target tissues, with EOMs typically undergoing a progressive transition from inflammatory edema to fat infiltration and fibrosis (5, 6). Therefore, accurately identifying diverse pathological changes in the EOMs is critical for tailoring effective personalized therapy strategies. However, pathological assessment of EOMs in TED patients faces inherent limitations: tissue sampling is only feasible during rehabilitative surgery in the stable phase, and pathological examination is exceptionally rare in clinical practice. This current situation underscores the clinical value of developing noninvasive imaging techniques for evaluating pathological changes in EOMs.

Previous studies have adopted a higher signal intensity ratio, apparent diffusion coefficient, and T2 relaxation time to represent EOM edema; a larger fat fraction to indicate fatty infiltration within EOMs; as well as a lower T1 relaxation time to reflect EOM fibrosis (7, 8, 9, 10, 11). However, these parameters only reflect a single pathological change with insufficient specificity. Although the first-order texture features reveal the heterogeneous manifestation of EOMs, they are unable to explain the exact causes (12). The ‘iterative decomposition of water and fat with echo asymmetric and least-squares estimation (IDEAL)’ technique effectively separates water and fat signals based on their different chemical shifts (13). Therefore, a comprehensive analysis of signal intensity (SI) features in water and fat images could visually reflect the distribution of water and fat contents, thus representing edematous and lipogenic lesions within EOMs.

Habitat imaging is a biologically validated approach for characterizing and quantifying tissue heterogeneity and was initially developed in oncologic imaging (14). Subsequent studies have extended this methodology to other disease contexts, such as the identification of high-risk vulnerable plaques in intracranial atherosclerosis using high-resolution vessel wall imaging, where compositional heterogeneity is closely linked to clinical risk stratification (15). TED exhibits significant heterogeneity in the pathological changes of EOMs. Texture analysis-based research presented a clear correlation between increased EOM heterogeneity and poor response to IVGC therapy (16). By combining habitat analysis with IDEAL imaging, we divided the EOMs into distinct subregions, referred to as habitats, based on the heterogeneous distribution of water and fat signal components within the EOMs. These habitats represent imaging-defined tissue compartments and provide a quantitative description of EOM heterogeneity related to edema and fatty infiltration. This noninvasive approach enables quantitative assessment of EOM heterogeneity and facilitates biological interpretation of the derived imaging parameters. To our knowledge, this is the first study to apply habitat analysis to TED.

By comparing the imaging parameters between responsive and unresponsive groups using IDEAL imaging-based habitat analysis across the treatment course, this study aims to (i) elucidate the heterogeneity in the EOMs and (ii) explore the characteristics of EOM remodeling.

## Materials and methods

### Study population

The study design and patient data usage comply with the Declaration of Helsinki. Additionally, Tongji Hospital, Tongji Medical College, and Huazhong University of Science and Technology approved this retrospective study and waived the requirement for informed consent (TJ-IRB202409032).

**Figure 1:**
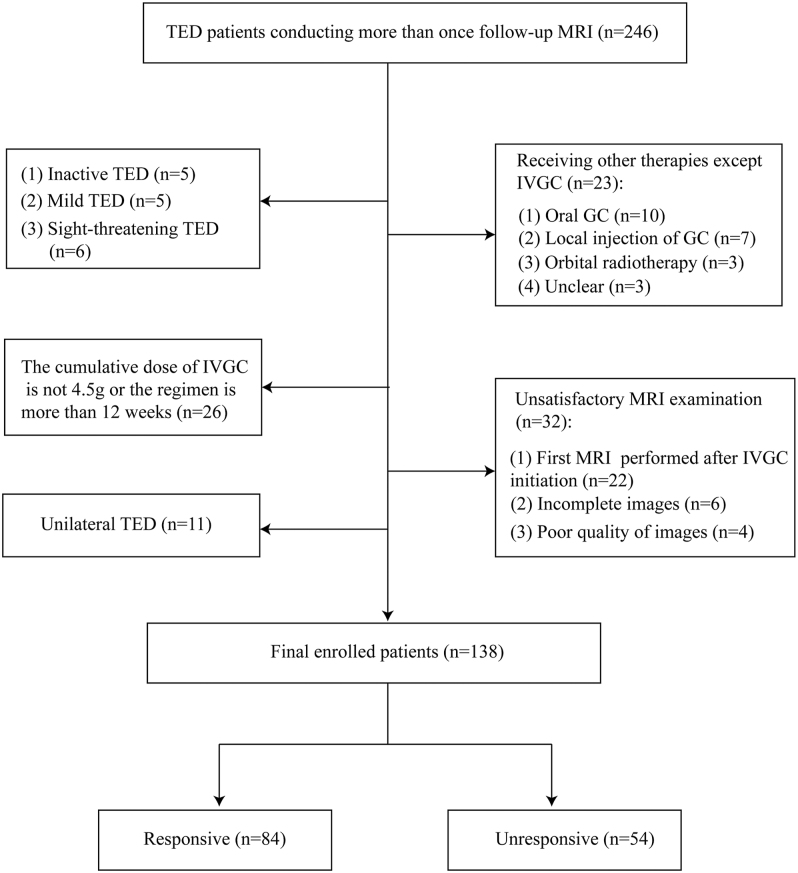
Flow chart of patients enrolling. IVGC, intravenous glucocorticoid; TED, thyroid eye disease.

We initially identified 246 TED patients with more than one follow-up MRI between March 2017 and July 2024. Inclusion criteria comprised (i) active and moderate-to-severe TED, (ii) EUGOGO-compliant IVGC therapy completion, (iii) analyzable pre-/post-treatment orbital MRI, (iv) 18–65 years age range, (v) ≤18 months disease duration, (vi) involvement of both eyes, (vii) received no prior treatments except IVGC, and (viii) no concurrent orbital disorders. We ultimately enrolled 138 patients (mean age: 46 ± 11 years; 55 men) clinically diagnosed with active and moderate-to-severe TED (Fig. 1). Disease activity and severity were assessed using the clinical activity score (CAS) and EUGOGO classification, respectively (3).

The clinical characteristics of the patients, including sex, age, smoking status, disease duration, proptosis, CAS, thyroid immune status, and thyroid function hormone levels, were documented. Thyroid function hormone levels were recorded separately within one week before treatment and within one week after treatment. The treatment regimen consisted of intravenous methylprednisolone administered at 0.5 g per week for 6 weeks, followed by 0.25 g per week for the subsequent 6 weeks, totaling a course of 12 weeks. Within two weeks after treatment, the therapeutic effect was evaluated by an experienced endocrinologist according to EUGOGO criteria (3). The criteria are defined by a set of fully objective metrics: a reduction in lid aperture of at least 2 mm, a decrease of at least 1 point in the five-item CAS (excluding spontaneous or gaze-evoked pain), a reduction in exophthalmos of at least 2 mm, and an increase in extraocular muscle duction of at least 8 degrees. A responsive therapeutic effect can be considered when at least two of these features show improvement in one eye, with no worsening observed in the other eye. Patients were then classified into responsive and unresponsive groups.

### MRI scanning

The initial and final MRI scans were performed using a 3T scanner (Discovery 750; GE Healthcare, USA) with a 32-channel head coil, conducted within one week before and after therapy, respectively. Patients were positioned supine with cotton balls in the ears for noise reduction, medical tape gently applied to maintain eyelid closure, and foam pads alongside the head to minimize motion.

Coronal T2 IDEAL parameters were as follows: echo time, 68 ms; repetition time, 2,200 ms; flip angle, 111°; matrix, 320 × 224; field of view, 20 cm; bandwidth, 63; number of excitations, 2; slice thickness, 3 mm; spacing, 0.6 mm; total slices, 15; and scan time, 2 min 52 s.

### Image segmentation and feature extraction

To segment the bilateral EOMs, 3D Slicer (https://www.slicer.org/) was used. The medial, inferior, lateral, and superior recti, along with the superior and inferior obliques, were manually delineated slice-by-slice on water images from the orbital apex to the posterior globe as integrated anatomical structures. The superior rectus and levator palpebrae were regarded as a complex due to their close anatomical relationship. Subsequently, the volume of interest masks for the EOMs were transferred to corresponding fat images and adjusted to better align with the muscle boundaries. Two neuroradiologists (seven and ten years of experience) independently performed the segmentation and feature extraction, with one reader repeating measurements at an interval of one month.

The original images and corresponding masks were imported into the MR station (Chengdu Zhongying Medical Technology Co., China) and processed with the MPQuan-Habitat module. The resampling served as a crucial preprocessing step to achieve precise spatial correspondence across multimodal imaging datasets, ensuring voxel-level registration accuracy. Habitat subregions were generated using the Otsu thresholding method, which is based on the image’s gray level histogram. The thresholding process is committed to seeking an optimal gray value, which satisfies maximal between-cluster variance and minimal within-cluster variance (17). The result is a binary image, where each voxel is classified as either white or black depending on whether its gray value is above or below the threshold, effectively dividing the image into two distinct regions.

**Figure 2:**
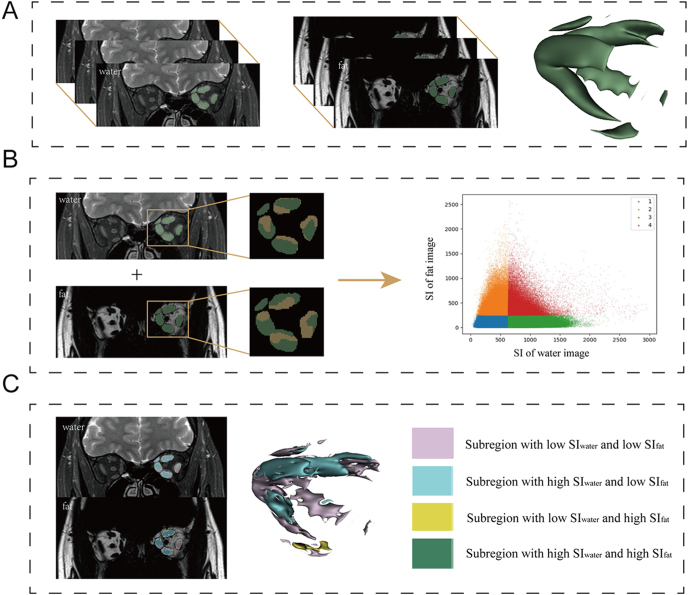
Workflow of image processing. (A) Segmentation of extraocular muscles in the left orbit. (B) Binary subregions produced in water and fat images using the Otsu method. (C) Four resulting subregions.

SI-based threshold values were established through volumetric delineation of all EOMs (four rectus and two oblique muscles) across consecutive orbital MRI layers in all included subjects. These thresholds were calculated separately for water and fat images, enabling subsequent segmentation of each image type into two distinct subregions based on their respective SI thresholds. The resulting subregions from each image were then combined to acquire four final subregions: low SI_water_ and low SI_fat_ (LL) region, high SI_water_ and low SI_fat_ (HL) region, low SI_water_ and high SI_fat_ (LH) region, and high SI_water_ and high SI_fat_ (HH) region (Fig. 2). The habitat features of EOMs, including the total volume, subregional volumes, and volume percentage (VP), were extracted for subsequent analysis. Changes in habitat parameters after therapy were expressed as Δ = post − pre, with percentage changes shown as Δ% = (post − pre)/pre × 100%.

### Statistical analysis

The statistical analyses were performed by SPSS (version 21; IBM Corp., USA). The repeatability between two different measurements was evaluated by the intraclass correlation coefficient (ICC) with a 95% confidence interval (CI). Pearson’s χ2 test was used to analyze the categorical parameters. For all continuous variables, the Shapiro–Wilk test was first used to identify the normality. Then, the data according to normal distribution were analyzed with Student’s *t*-test and expressed as mean ± standard deviation; otherwise, the Mann–Whitney U test was conducted, and the data were displayed as median (interquartile range). Binary logistic analysis and receiver operating characteristic (ROC) curve analysis were used to determine the valuable parameters for evaluating the therapeutic effect. *P* < 0.05 was considered a statistically significant difference. The Pearson or Spearman’s correlation coefficient was applied to normally or non-normally distributed data, respectively. The absolute value of the correlation coefficient (*r*) was interpreted as follows: poor (*r* < 0.40), moderate (*r* = ≥0.40 – < 0.60), good (*r *= ≥0.60 – < 0.80), and excellent (*r* ≥ 0.80).

## Results

### Subjects in the study population

Patients were stratified into two groups based on treatment response: responsive (*n* = 84) and unresponsive (*n* = 54). There were differences in disease duration (*P* = 0.003), post-CAS (*P* < 0.001), post-thyroid-stimulating hormone levels (*P* = 0.03), and post-thyroid-stimulating hormone receptor antibody levels (*P* = 0.008) between responsive and unresponsive groups. There were no significant differences in other clinical characteristics (all *P* > 0.05). Detailed characteristics of the study population are presented in Table 1.

**Table 1:** Clinical characteristics of patients with different therapeutic responses. Parameters are presented as median (interquartile range) or as *n* (%).

	Responsive (*n* = 84)	Unresponsive (*n* = 54)	*P*
Age (years)	49 (40, 52)	47 (42, 54)	0.95
Sex			0.40
Male	36 (42.86%)	19 (35.19%)	
Female	48 (57.14%)	35 (64.81%)	
Smoking			0.54
Yes	13 (15.48%)	11 (20.37%)	
No	71 (84.52%)	43 (79.63%)	
Disease duration (months)	5 (3, 8)	6 (6, 10)	0.003^†^
Pre-proptosis	21.10 (19.20, 23.35)	19.95 (18.50, 22.78)	0.18
Post-proptosis	21.00 (19.00, 22.70)	20.90 (19.55, 23.85)	0.34
Pre-CAS	3 (2, 4)	3 (2, 3)	0.18
Post-CAS	1 (1, 1)	3 (3, 3)	<0.001^†^
Thyroid immune status			0.60
Hyperthyroidism	59 (70.24%)	43 (79.63%)	
Euthyroidism	12 (14.29%)	6 (11.11%)	
Hypothyroidism	13 (15.47%)	5 (9.26%)	
Thyroid function hormone levels*			
Pre-TSH (μIU/mL)	0.76 (0.05, 3.56)	0.28 (0.01, 1.93)	0.07
Pre-T3 (pg/mL)	3.14 (2.60, 3.88)	3.09 (2.70, 4.17)	0.92
Pre-T4 (ng/L)	12.38 (8.66, 14.62)	12.56 (10.24, 13.67)	0.66
Pre-TgAb (IU/mL)	33.39 (10.00, 183.07)	28.79 (10.41, 183.07)	0.87
Pre-TPOAb (IU/mL)	28.34 (9.79, 157.10)	25.89 (13.14, 106.08)	0.94
Pre-TRAb (IU/L)	9.15 (3.36, 18.79)	12.79 (7.31, 20.40)	0.37
Post-TSH (μIU/mL)	2.66 (1.32, 4.53)	1.75 (0.35, 2.67)	0.03^†^
Post-T3 (pg/mL)	2.91 (2.58, 2.97)	3.00 (2.50, 4.04)	0.12
Post-T4 (ng/L)	10.31 (8.96, 10.31)	10.77 (8.57, 11.68)	0.79
Post-TgAb (IU/mL)	155.59 (12.71, 189.79)	79.18 (10.80, 100.33)	0.06
Post-TPOAb (IU/mL)	76.14 (11.06, 76.14)	38.89 (10.58, 49.12)	0.06
Post-TRAb (IU/L)	5.76 (2.03, 5.82)	9.55 (2.03, 9.82)	0.008^†^

* ‘Pre-’ and ‘post-’ denote the time points before and after IVGC therapy, respectively.

^†^ Indicates a statistically significant result at *P* < 0.05.

CAS, clinical activity score; IVGC, intravenous glucocorticoid; TSH, thyroid-stimulating hormone; T3, free triiodothyronine; T4, free thyroxine; TgAb, thyroglobulin antibody; TPOAb, thyroid peroxidase antibody; TRAb, thyroid-stimulating hormone receptor antibody.

### Differences in habitat features between responders and non-responders

The intra-observer and inter-observer reproducibility of habitat feature measurements demonstrated excellent reliability, as evidenced by an ICC (95% CI) of 0.95 (0.92–0.97) and 0.91 (0.87–0.93), respectively. At baseline, the responsive group demonstrated significantly larger whole-region volume compared to unresponsive patients (*P* = 0.004). Habitat analysis further revealed that responders had greater volume and VP in the HL subregion but lower VP in the LL subregion (all *P* < 0.05). Following therapy, no significant intergroup differences were observed in the whole region or the HL subregion. However, responders exhibited notably larger volume and VP in the LH subregion, alongside smaller volume and VP in the LL subregion (all *P* < 0.05). Longitudinal analysis demonstrated that responders showed a significant reduction in whole EOM volume, whereas non-responders exhibited an overall increase. In addition, responders displayed more pronounced decreases in the HL subregion, consistent with edema reduction, and greater increases in the LH subregion, suggestive of progressive myosteatosis or tissue remodeling, across both absolute (Δ) and relative (Δ%) volumetric change metrics (all *P* < 0.05). The detailed results are shown in Tables 2 and 3.

**Table 2:** Comparison of habitat parameters of EOMs in TED patients between responsive and unresponsive groups. Parameters are presented as median (interquartile range).

Parameters*	Responsive (*n* = 84)	Unresponsive (*n* = 54)	*P*
Whole region			
Pre-volume (mL)	3.79 (2.89, 4.54)	3.05 (2.29, 4.18)	0.004^†^
Post-volume (mL)	3.36 (2.75, 4.12)	3.39 (2.60, 4.93)	0.43
LL region			
Pre-volume (mL)	1.40 (0.87, 3.13)	1.55 (1.07, 3.26)	0.16
Post-volume (mL)	1.67 (1.21, 2.80)	2.26 (1.25, 4.00)	0.04^†^
Pre-VP (%)	43.03 (24.92, 94.02)	67.35 (38.26, 95.03)	0.02^†^
Post-VP (%)	55.69 (38.44, 86.46)	83.70 (40.48, 95.35)	0.01^†^
HL region			
Pre-volume (mL)	0.93 (0.06, 2.22)	0.46 (0.03, 1.14)	0.009^†^
Post-volume (mL)	0.28 (0.00, 1.04)	0.20 (0.00, 1.00)	0.41
Pre-VP (%)	32.64 (1.62, 54.74)	14.13 (0.79, 35.93)	0.008^†^
Post-VP (%)	9.85 (0.00, 30.88)	5.23 (0.00, 28.64)	0.33
LH region			
Pre-volume (mL)	0.21 (0.12, 0.39)	0.27 (0.13, 0.40)	0.59
Post-volume (mL)	0.45 (0.28, 0.75)	0.27 (0.13, 0.45)	<0.001^†^
Pre-VP (%)	5.72 (2.87, 10.44)	8.18 (3.82, 12.50)	0.11
Post-VP (%)	13.83 (7.75, 22.94)	7.68 (3.42, 14.51)	<0.001^†^
HH region			
Pre-volume (mL)	0.13 (0.00, 0.44)	0.04 (0.00, 0.27)	0.07
Post-volume (mL)	0.10 (0.00, 0.28)	0.01 (0.00, 0.17)	0.08
Pre-VP (%)	4.12 (0.00, 10.42)	1.34 (0.00, 9.06)	0.17
Post-VP (%)	2.96 (0.00, 9.01)	0.19 (0.00, 6.29)	0.11

* ‘Pre-’ and ‘post-’ denote the time points before and after IVGC therapy, respectively.

^†^ Indicates a statistically significant result at *P* < 0.05.

IVGC, intravenous glucocorticoid; LL, low SI_water_ and low SI_fat_; HL, high SI_water_ and low SI_fat_; LH, low SI_water_ and high SI_fat_; HH, high SI_water_ and high SI_fat_; SI, signal intensity; EOM, extraocular muscle; TED, thyroid eye disease; VP, volume percentage.

**Table 3:** Comparison of the changes in habitat features of EOMs in TED patients between responsive and unresponsive groups. Parameters are presented as median (interquartile range).

Parameters	Responsive (*n* = 84)	Unresponsive (*n* = 54)	*P*
Whole region			
Δ volume (mL)	−0.39 (−0.81, 0.10)	0.42 (0.01, 0.90)	<0.001*
Δ% volume (%)	−10.31 (−20.49, 3.26)	13.79 (0.59, 30.45)	<0.001*
LL region			
Δ volume (mL)	0.16 (−0.53, 0.59)	0.26 (−0.16, 0.87)	0.03*
Δ% volume (%)	10.92 (−19.50, 65.99)	12.08 (−9.35, 40.69)	0.85
Δ VP (%)	2.25 (−3.71, 20.03)	−0.49 (−5.94, 3.48)	0.06
Δ% VP (%)	5.15 (−4.33, 77.61)	−0.52 (−10.19, 8.28)	0.01*
HL region			
Δ volume (mL)	−0.22 (−1.13, 0.00)	0.00 (−0.21, 0.26)	<0.001*
Δ% volume (%)	−43.70 (−76.09, 0.00)	0.00 (−65.16, 50.68)	<0.001*
Δ VP (%)	−7.57 (−26.75, 0.00)	0.00 (−7.38, 3.37)	<0.001*
Δ% VP (%)	−40.41 (−72.50, 0.00)	0.00 (−64.29, 33.02)	0.002*
LH region			
Δ volume (mL)	0.21 (0.08, 0.37)	0.04 (−0.03, 0.13)	<0.001*
Δ% volume (%)	88.67 (39.00, 186.60)	19.61 (−16.86, 70.97)	<0.001*
Δ VP (%)	6.46 (2.31, 12.62)	0.48 (−1.81, 3.32)	<0.001*
Δ% VP (%)	115.90 (42.58, 228.62)	7.35 (−38.72, 51.20)	<0.001*
HH region			
Δ volume (mL)	0.00 (−0.13, 0.01)	0.00 (−0.01, 0.05)	0.06
Δ% volume (%)	0.00 (−43.61, 5.90)	0.00 (−31.63, 34.81)	0.35
Δ VP (%)	0.00 (−2.83, 0.94)	0.00 (−1.01, 1.04)	0.42
Δ% VP (%)	0.00 (−37.15, 13.29)	0.00 (−28.22, 16.69)	0.97

* Indicates a statistically significant result at *P* < 0.05.

LL, low SI_water_ and low SI_fat_; HL, high SI_water_ and low SI_fat_; LH, low SI_water_ and high SI_fat_; HH, high SI_water_ and high SI_fat_; SI, signal intensity; EOM, extraocular muscle; TED, thyroid eye disease; VP, volume percentage; Δ, variation of parameter after therapy; Δ%, rate of variation in parameter.

### Performance of habitat parameters for evaluating the therapeutic effect of IVGC

**Figure 3:**
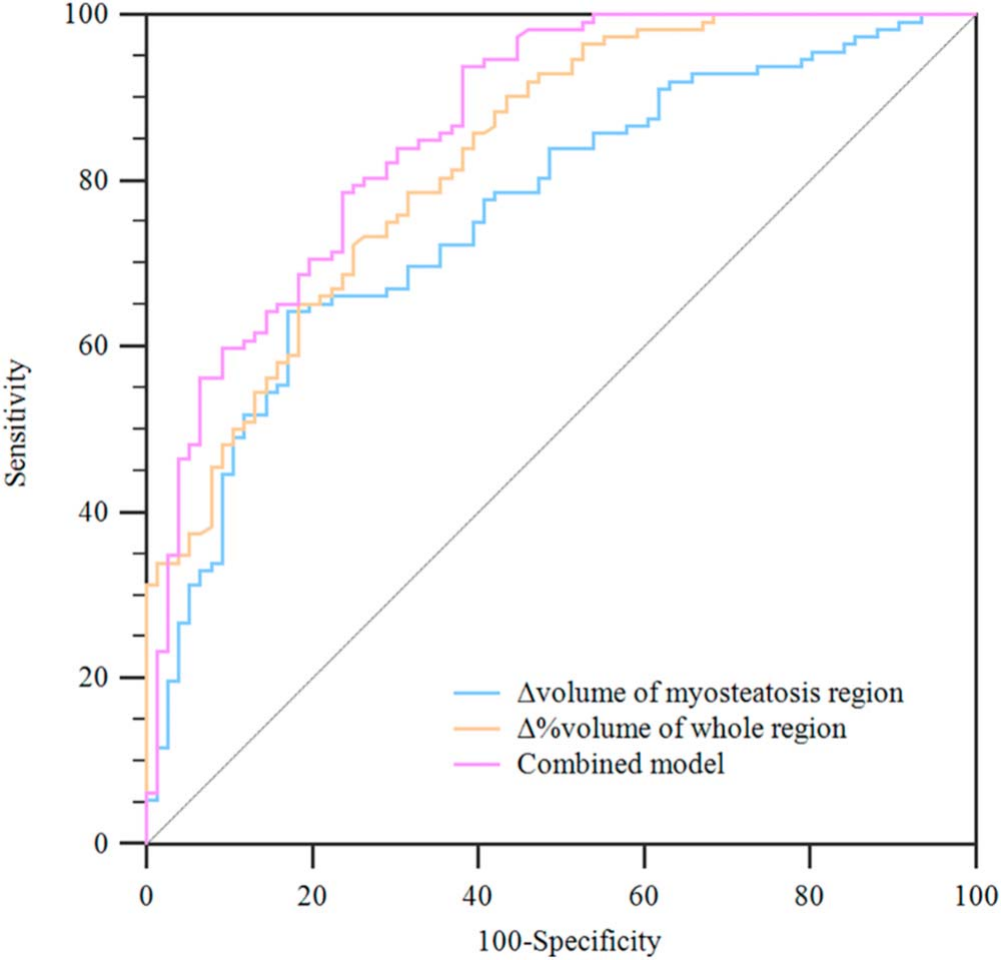
ROC curve showing the efficiency of valuable habitat parameters in evaluating IVGC. IVGC, intravenous glucocorticoid; ROC, receiver operating characteristic.

Based on the significant habitat parameters obtained from univariable analysis, binary logistic analysis showed that the Δ volume of the LH region (*P* < 0.001) and Δ% volume of the whole region (*P* < 0.001) were the most valuable parameters for evaluating the therapeutic effect of IVGC therapy. ROC curve analysis revealed that the Δ volume of the LH region generated an area under the ROC curve (AUC) of 0.76, cutoff value of 0.15 mL, sensitivity of 64.29%, and specificity of 82.89%; the Δ% volume of the whole region yielded an AUC of 0.83, cutoff value of 1.97%, sensitivity of 72.32%, and specificity of 75.00%; and their combination achieved an AUC of 0.86, sensitivity of 93.75%, and specificity of 61.84% (Table 4 and Fig. 3).

**Table 4:** Efficiency of valuable habitat parameters in evaluating IVGC in TED patients. The cutoff values for calculating sensitivity and specificity were those that yielded the largest Youden index value (sensitivity + specificity − 1).

Parameters	Cutoff	AUC (95%CI)	Sensitivity	Specificity	*P*
Δ volume of myosteatosis region	>0.15 mL	0.76 (0.70, 0.82)	64.29%	82.89%	<0.001*
Δ% volume of whole region	≤1.97%	0.83 (0.77, 0.88)	72.32%	75.00%	<0.001*
Combined model	-	0.86 (0.81, 0.91)	93.75%	61.84%	<0.001*

* Indicates a statistically significant result at *P* < 0.05.

AUC, area under the curve; CI, confidence interval; IVGC, intravenous glucocorticoid; TED, thyroid eye disease; Δ, variation of parameter after therapy; Δ%, rate of variation in parameter.

### Difference of habitat parameters before and after IVGC therapy in patients of the two groups

**Figure 4:**
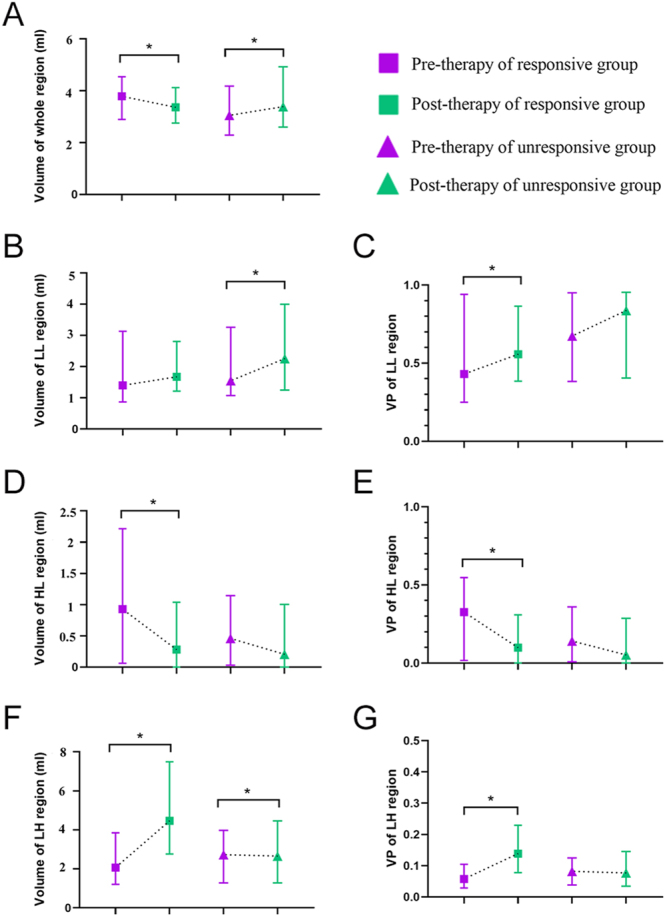
Plots showing the comparison of habitat parameters in responsive and unresponsive groups between pre- and post-therapy. The habitat parameters include (A) volume of the whole region, (B) volume of the LL region, (C) VP of the LL region, (D) volume of the HL region, (E) VP of the HL region, (F) volume of the LH region, and (G) VP of the LH region. LL, low SI_water_ and low SI_fat_; HL, high SI_water_ and low SI_fat_; LH, low SI_water_ and high SI_fat_; SI, signal intensity; and VP, volume percentage. The asterisk indicates that the results are significantly different.

In the responsive group, the volume of the whole region, along with the volume and VP of the HL region, decreased significantly after therapy (all *P* < 0.001). Moreover, the VP of the LL region (*P* = 0.001) as well as the volume (*P* < 0.001) and VP (*P* < 0.001) of the LH region increased significantly after therapy. In regard to the unresponsive group, the volume of the whole region (*P* < 0.001), along with the volume of the LL region (*P* < 0.001) and the LH region (*P* = 0.005), increased after IVGC (Fig. 4).

### Variances of features between the LL region and the LH region in TED patients

**Figure 5:**
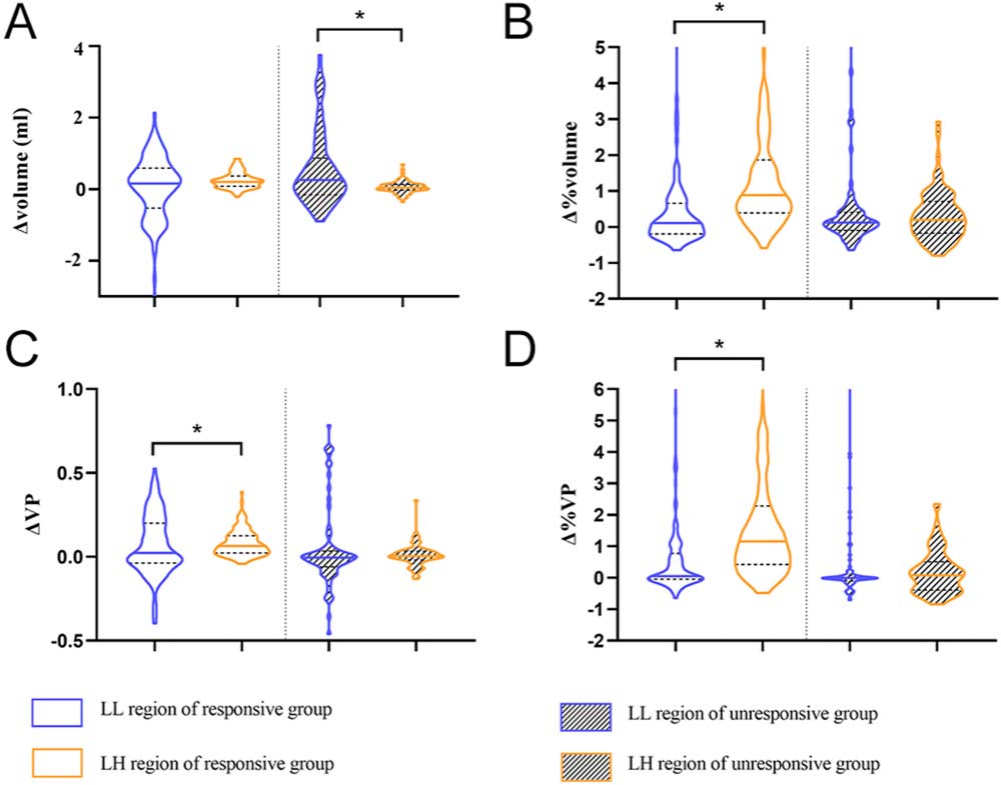
Violin plots showing the comparison of habitat parameters in both groups between the LL and LH regions. The habitat parameters consist of (A) the variation of volume after therapy (Δ volume), (B) the rate of variation in volume (Δ% volume), (C) Δ VP, and (D) Δ% VP. LL, low SI_water_ and low SI_fat_; LH, low SI_water_ and high SI_fat_; SI, signal intensity; VP, volume percentage. The asterisk indicates that the results are significantly different.

In the responsive group, Δ VP (6.46 vs 2.25%, *P* = 0.05), Δ% volume (88.67 vs 10.92%, *P* < 0.001), and Δ% VP (115.90 vs 5.15%, *P* < 0.001) in the LH region were significantly larger than those in the LL region. Collectively, these findings indicate that treatment-related EOM remodeling in responsive patients is primarily characterized by fatty infiltration. In contrast, the Δ volume (0.26 vs 0.04 mL, *P* = 0.009) of the LL region was greater than that of the LH region in the unresponsive group, suggesting that treatment-related EOM remodeling in these patients may be predominantly fibrotic in nature (Fig. 5).

### Correlations between clinical characteristics and habitat features in TED patients

**Figure 6:**
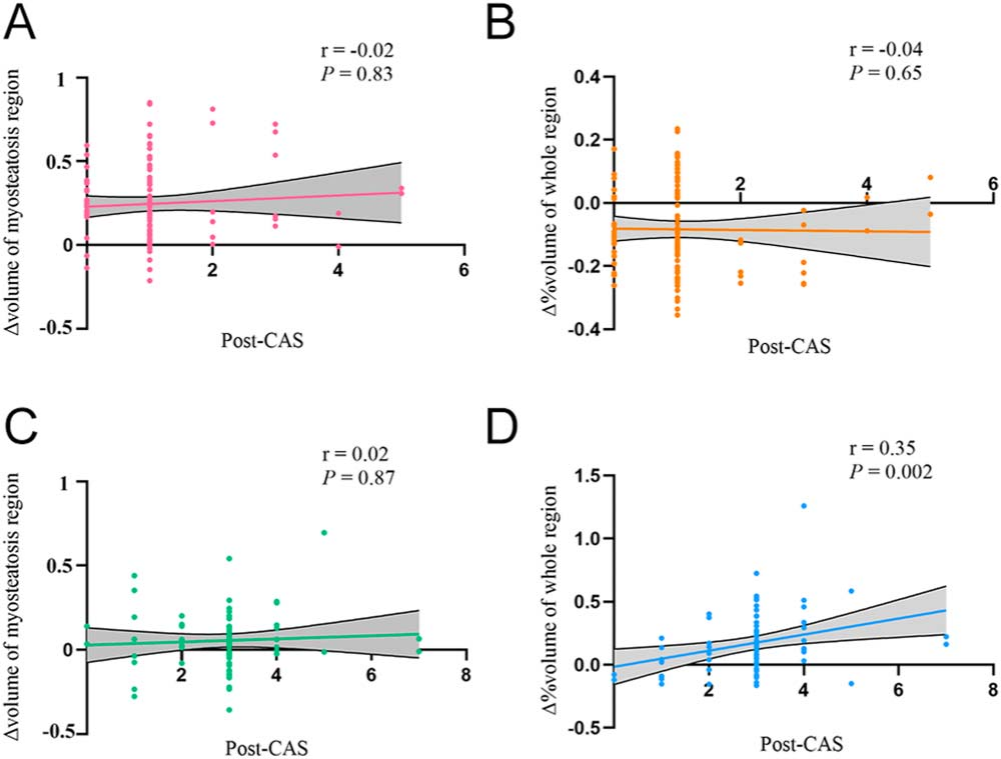
Scatter plots showing the correlations between the post-CAS and valuable habitat parameters in responders (A and B) and non-responders (C and D). CAS, clinical activity score.

Correlation analysis demonstrated that post-CAS showed no significant association with Δ volume of the LH region (responders: *r* = −0.02, *P* = 0.83 and non-responders: *r* = 0.02, *P* = 0.87) or Δ% volume of the whole region in responders (*r* = −0.04, *P* = 0.65). However, in non-responders, post-CAS exhibited a weak but statistically significant positive correlation with Δ% volume of the whole region (*r* = 0.35, *P* = 0.002), as illustrated in Fig. 6.

## Discussion

There were three main findings in this study: (i) IDEAL imaging-based habitat analysis could noninvasively represent the pathologic changes of edema and myosteatosis within EOMs of TED patients; (ii) after IVGC therapy, TED patients exhibited a remodeling process from edema to myosteatosis in EOMs, particularly evident in responders; and (iii) the combination of Δ volume of myosteatosis region and Δ% volume of the whole region achieved optimal performance in evaluating the therapeutic effect of IVGC.

Prior imaging parameters reflecting heterogeneity lack interpretability, making them difficult to apply in clinical practice. As an emerging biological validation method, habitat imaging holds great promise for revealing the biological significance underlying imaging signatures (18). IDEAL imaging-based habitat analysis noninvasively captures pathological changes by identifying their respective SI characteristics in water and fat images. Therefore, this method facilitates a deeper understanding of the mechanisms underlying heterogeneous changes in the EOMs. In addition, longitudinal observation of habitat features throughout the treatment process allows for the visualization of microscopic changes and remodeling patterns within the EOMs, enabling precise evaluation of the therapeutic effect.

The extraction of habitat features allowed the recognition of an active region (high SI_water_) within EOMs. The muscle biopsy of the lateral rectus showed that the SI ratio of the water image was positively correlated with the glycosaminoglycan-volume fraction in TED (19). Therefore, we regarded the HL region as an edematous lesion, characterized by excessive accumulation of hydrophilic glycosaminoglycans resulting from immune cell infiltration and cytokine mediation during the active phase of TED (20). Previous studies have utilized water fraction derived from T2 IDEAL and other imaging parameters, such as T2 relaxation time, to indirectly quantify the degree of edema in the EOMs (21, 22). The present habitat analysis not only confirmed that responders exhibited more edematous EOMs but also intuitively demonstrated the distribution and quantification of the edema region. After IVGC treatment, the habitat subregion of edema in responders significantly decreased, while no noticeable changes were observed in non-responders. These observations align with the established understanding that TED patients with more severe orbital soft tissue inflammation demonstrate greater clinical benefits from IVGC therapy (3, 23). Notably, the HH region occupied the smallest proportion, localized at the water–fat interface between EOMs and orbital fat, without relevant clinical significance.

The subregions exhibiting low SI_water_ likely correspond to inactive tissue components within the EOMs. Das *et al.* (9) pioneered quantitative fat fraction measurement (SI_fat_/(SI_water_ + SI_fat_)) in EOMs of TED and suggested that the increased fat fraction was consistent with adipogenesis within the EOMs, reflecting chronicity of the disease. Hence, we defined the LH region as demonstrating high myosteatosis similarity, reflecting adipogenic differentiation of resident fibro/adipogenic progenitors under pathological conditions, including muscle disuse and glucocorticoid exposure (24). Fibrosis refers to the formation of permanent scar tissue resulting from the overabundance of dense extracellular matrix components, primarily collagen, containing neither water nor fat components (25). Combining SI ratio (≤2.17) and extracellular volume fraction (>48.1%) thresholds effectively detected EOM fibrosis in inactive TED, with histopathologically confirmed correlation to collagen deposition (19). Therefore, we consider the fibrotic changes within EOMs conform to low SI_water_ and low SI_fat_. In addition, the treated EOMs in the inflammatory resolution phase exhibit significantly reduced SI_water_. If not yet progressed to the chronic quiescent phase, they maintain similarly low SI_fat_. Notably, this imaging feature overlaps with the aforementioned fibrotic EOMs, necessitating further analytical methods for definitive differentiation.

The resulting habitat features directly depicted the changing tendency from the HL region to the LH and LL regions after IVGC in TAO patients, with responders mainly exhibiting increased LH region and non-responders largely showing increased LL region. The regions displaying LH and LL are essentially part of the inflammation resolution process. In other words, patients with varying therapeutic responses could still derive some benefit from IVGC therapy, with the key difference being whether they achieved clinical remission. Additionally, it may deepen our comprehension of the progression and outcome of TED, further suggesting that myosteatosis may indicate a more favorable prognosis. Several studies have demonstrated the value of radiomics analysis in predicting the treatment response to IVGC (26, 27). However, the lack of interpretability of radiomic signatures limits their clinical application. The visualization of habitat subregions facilitates the precise evaluation of pathological changes in each EOM. Our habitat analysis demonstrated that Δ% volume of the whole region showed a significant positive correlation with post-CAS, while Δ volume of the myosteatosis region served as a valuable biomarker for evaluating therapeutic effect. The post-CAS showed no significant correlation with Δ volume of the myosteatosis region in TED patients, indicating that myosteatosis may persist or progress independently of active inflammation and is thus not adequately reflected by CAS assessment. These findings further highlighted the advantage of habitat analysis in interpreting imaging features. The final valuable habitat parameters have the potential to replenish the therapeutic efficacy assessment system. Moreover, the adoption of habitat imaging in routine clinical practice could enhance diagnostic precision and enable more accurate monitoring of TED progression and treatment response.

Several limitations should be acknowledged in this study. First, as a retrospective single-center study with a limited sample size, the generalizability of our findings may be constrained. Further validation through larger-scale, multicenter prospective studies would be required to substantiate these preliminary observations. Second, we explored the orbital tissues’ remodeling only in a short period. The absence of long-term follow-up restricts our ability to assess the persistence of tissue changes and their lasting impact on clinical outcomes. Third, we only analyzed basic morphological parameters of EOMs, which, although informative, may not capture the full spectrum of tissue changes that those other advanced features could reveal. Fourth, the Otsu algorithm was limited in identifying fibrotic regions of EOMs, requiring more sophisticated habitat analysis methods for accurate differentiation. Fifth, histological analysis of EOMs remains challenging to obtain in clinical practice. Consequently, the precise pathological characteristics of EOMs in TED require further elucidation. Future studies correlating imaging parameters with histopathological changes would provide valuable insights into disease mechanisms.

In conclusion, IDEAL-based habitat imaging analysis holds significant promise for visualizing edema and myosteatosis in the heterogeneous EOMs of TED patients. The findings of this study not only exhibited the difference in EOM remodeling after IVGC between the responsive group and unresponsive group but also demonstrated the value of myosteatosis in evaluating the therapeutic effect of IVGC. The visualization and quantification of EOM heterogeneity could bridge the gap between macroscopic disease status and microscopic pathological changes. This approach contributes to the evaluation of disease activity and severity, while offering potential clinical utility for TED management decisions.

## Declaration of interest

The authors declare that there is no conflict of interest that could be perceived as prejudicing the impartiality of the study reported.

## Funding

This study received funding from the National Natural Science Foundation of Chinahttps://doi.org/10.13039/501100001809 (No. 82471967).

## Author contribution statement

LZ undertook data curation, formulated the methodology, and prepared the original manuscript. YY performed data curation, executed validation procedures, and prepared the original manuscript. YC, WL, and FL conducted formal analysis, developed the software, and advanced the methodological framework. QW conceptualized the study, oversaw data curation, and administered the project. GY facilitated data curation, contributed to methodological refinement, and carried out validation. JZ conceptualized the study, directed project administration, and oversaw manuscript preparation, review, and editing. All authors read and approved the final manuscript.

## References

[1] Bahn RS. Graves’ ophthalmopathy. N Engl J Med 2010 362 726–738. (10.1056/nejmra0905750) 20181974 PMC3902010

[2] Kulbay M, Tanya SM, Tuli N, et al. A comprehensive review of thyroid eye disease pathogenesis: from immune dysregulations to novel diagnostic and therapeutic approaches. Int J Mol Sci 2024 25 11628. (10.3390/ijms252111628) 39519180 PMC11546489

[3] Bartalena L, Kahaly GJ, Baldeschi L, et al. The 2021 European Group on Graves’ orbitopathy (EUGOGO) clinical practice guidelines for the medical management of Graves’ orbitopathy. Eur J Endocrinol 2021 185 G43–G67. (10.1530/eje-21-0479) 34297684

[4] Vandewalle J, Luypaert A, De Bosscher K, et al. Therapeutic mechanisms of glucocorticoids. Trends Endocrinol Metab 2018 29 42–54. (10.1016/j.tem.2017.10.010) 29162310

[5] Rundle FF. Management of exophthalmos and related ocular changes in Graves’ disease. Metabolism 1957 6 36–48. 13386967

[6] Kaichi Y, Tanitame K, Itakura H, et al. Orbital fat volumetry and water fraction measurements using T2-weighted FSE-IDEAL imaging in patients with thyroid-associated orbitopathy. AJNR Am J Neuroradiol 2016 37 2123–2128. (10.3174/ajnr.a4859) 27365323 PMC7963797

[7] Mayer EJ, Fox DL, Herdman G, et al. Signal intensity, clinical activity and cross-sectional areas on MRI scans in thyroid eye disease. Eur J Radiol 2005 56 20–24. (10.1016/j.ejrad.2005.03.027) 15896938

[8] Politi LS, Godi C, Cammarata G, et al. Magnetic resonance imaging with diffusion-weighted imaging in the evaluation of thyroid-associated orbitopathy: getting below the tip of the iceberg. Eur Radiol 2014 24 1118–1126. (10.1007/s00330-014-3103-3) 24519110

[9] Das T, Roos JCP, Patterson AJ, et al. T2-relaxation mapping and fat fraction assessment to objectively quantify clinical activity in thyroid eye disease: an initial feasibility study. Eye 2019 33 235–243. (10.1038/s41433-018-0304-z) 30538310 PMC6367394

[10] Liu P, Chen L, Wang QX, et al. Histogram analysis of T2 mapping for detecting early involvement of extraocular muscles in patients with thyroid-associated ophthalmopathy. Sci Rep 2020 10 19445. (10.1038/s41598-020-76341-6) 33173086 PMC7655798

[11] Zhu H, Zou M, Wu D, et al. Quantitative assessment of extraocular muscles in Graves’ ophthalmopathy using T1 mapping. Eur Radiol 2023 33 9074–9083. (10.1007/s00330-023-09931-3) 37466707

[12] Zhai L, Wang Q, Liu P, et al. T2 mapping with and without fat-suppression to predict treatment response to intravenous glucocorticoid therapy for thyroid-associated ophthalmopathy. Korean J Radiol 2022 23 664–673. (10.3348/kjr.2021.0627) 35555881 PMC9174502

[13] Reeder SB, Pineda AR, Wen Z, et al. Iterative decomposition of water and fat with echo asymmetry and least-squares estimation (IDEAL): application with fast spin-echo imaging. Magn Reson Med 2005 54 636–644. (10.1002/mrm.20624) 16092103

[14] Robert A, Gatenby OG & Gillies RJ. Quantitative imaging in cancer evolution and ecology. Radiology 2013 269 8–14. (10.1148/radiol.13122697) 24062559 PMC3781355

[15] Gao Y, Li Z, Zhai X, et al. MRI-based habitat radiomics combined with vision transformer for identifying vulnerable intracranial atherosclerotic plaques and predicting stroke events: a multicenter, retrospective study. EClinicalMedicine 2025 82 103186. (10.1016/j.eclinm.2025.103186) 40235946 PMC11999680

[16] Wang YY, Wu Q, Chen L, et al. Texture analysis of orbital magnetic resonance imaging for monitoring and predicting treatment response to glucocorticoids in patients with thyroid-associated ophthalmopathy. Endocr Connect 2021 10 676–684. (10.1530/ec-21-0162) 34077388 PMC8240707

[17] Liu Y, Wang P, Wang S, et al. Heterogeneity matching and IDH prediction in adult-type diffuse gliomas: a DKI-based habitat analysis. Front Oncol 2023 13 1202170. (10.3389/fonc.2023.1202170) 38090477 PMC10713834

[18] Tomaszewski MR & Gillies RJ. The biological meaning of radiomic features. Radiology 2021 298 505–516. (10.1148/radiol.2021202553) 33399513 PMC7924519

[19] Ma R, Geng Y, Gan L, et al. Quantitative T1 mapping MRI for the assessment of extraocular muscle fibrosis in thyroid-associated ophthalmopathy. Endocrine 2022 75 456–464. (10.1007/s12020-021-02873-0) 34549377

[20] Davies TF, Andersen S, Latif R, et al. Graves’ disease. Nat Rev Dis Primers 2020 6 52. (10.1038/s41572-020-0184-y) 32616746

[21] Liu P, Luo B, Chen L, et al. Baseline volumetric T2 relaxation time histogram analysis: can it be used to predict the response to intravenous methylprednisolone therapy in patients with thyroid-associated ophthalmopathy? Front Endocrinol 2021 12 614536. (10.3389/fendo.2021.614536) PMC7947366 33716970

[22] Zhai L, Luo B, Wu H, et al. Prediction of treatment response to intravenous glucocorticoid in patients with thyroid-associated ophthalmopathy using T2 mapping and T2 IDEAL. Eur J Radiol 2021 142 109839. (10.1016/j.ejrad.2021.109839) 34252869

[23] Langericht J, Kramer I & Kahaly GJ. Glucocorticoids in Graves’ orbitopathy: mechanisms of action and clinical application. Ther Adv Endocrinol Metab 2020 11 2042018820958335. (10.1177/2042018820958335) 33403097 PMC7745544

[24] Hamrick MW, McGee-Lawrence ME & Frechette DM. Fatty infiltration of skeletal muscle: mechanisms and comparisons with bone marrow adiposity. Front Endocrinol 2016 7 69. (10.3389/fendo.2016.00069) PMC4913107 27379021

[25] Mann CJ, Perdiguero E, Kharraz Y, et al. Aberrant repair and fibrosis development in skeletal muscle. Skelet Muscle 2011 1 21. (10.1186/2044-5040-1-21) 21798099 PMC3156644

[26] Hu H, Chen L, Zhang JL, et al. T(2)-weighted MR imaging-derived radiomics for pretreatment determination of therapeutic response to glucocorticoid in patients with thyroid-associated ophthalmopathy: comparison with semiquantitative evaluation. J Magn Reson Imaging 2022 56 862–872. (10.1002/jmri.28088) 35092642

[27] Zhang H, Jiang M, Chan HC, et al. Whole-orbit radiomics: machine learning-based multi- and fused- region radiomics signatures for intravenous glucocorticoid response prediction in thyroid eye disease. J Transl Med 2024 22 56. (10.1186/s12967-023-04792-2) 38218934 PMC10787992

